# A lightweight CNN–transformer hybrid architecture with channel attention for real-time hazardous acoustic event detection

**DOI:** 10.3389/frai.2026.1824067

**Published:** 2026-06-02

**Authors:** Aigerim Altayeva, Nurzhan Omarov

**Affiliations:** 1 International Information Technology University, Almaty, Kazakhstan; 2 Al-Farabi Kazakh National University, Almaty, Kazakhstan

**Keywords:** acoustic events, audio analysis, classification, deep learning, detection, hazardous sounds, mel-spectrogram analysis, transformers

## Abstract

**Introduction:**

Hazardous acoustic event detection is critically important for intelligent surveillance, emergency response systems, and public safety monitoring applications. Accurate and real-time identification of dangerous sound events such as explosions, alarms, screaming, and weapon-related sounds can significantly improve situational awareness and accelerate emergency response in safety-critical environments.

**Methods:**

This study proposes a lightweight deep learning architecture for hazardous sound classification based on convolutional feature extraction and channel attention mechanisms. The proposed framework utilizes log-mel spectrogram representations as input and incorporates a TinyCNN backbone enhanced with squeeze-and-excitation channel attention modules to improve discriminative spectral feature learning while preserving computational efficiency. A custom balanced dataset consisting of eight hazardous acoustic classes, including crying, dog barking, emergency alarm, explosion, fire, glass breaking, screaming, and weapon-related sounds, was constructed with one thousand audio samples per class. The model was evaluated using accuracy, precision, recall, and F1-score metrics.

**Results:**

Experimental results demonstrate that the proposed architecture achieves strong multi-class classification performance while maintaining real-time inference capability suitable for edge deployment scenarios. Quantitative evaluations confirm the effectiveness of the lightweight framework for hazardous acoustic event detection. Additional ablation studies indicate that the integration of channel attention mechanisms and spectrogram-based augmentation strategies substantially improves model robustness, feature discrimination, and generalization performance.

**Discussion:**

The obtained findings demonstrate that the proposed lightweight channel-attention-enhanced architecture provides an efficient and reliable solution for real-time hazardous sound detection in intelligent monitoring and public safety systems. The combination of computational efficiency and robust classification performance highlights the suitability of the proposed framework for deployment in resource-constrained and edge-based environments.

## 1 Introduction

The rapid growth of intelligent sensing technologies and ubiquitous computing has intensified the demand for automated systems capable of recognizing hazardous acoustic events in real time. Environmental sound analysis plays a critical role in numerous safety-critical domains, including urban surveillance, smart homes, emergency response systems, and industrial monitoring. Detecting dangerous acoustic signals such as explosions, gunshots, glass breaking, or distress screams can significantly enhance situational awareness and enable timely interventions. Traditional monitoring solutions often rely on human observation or threshold-based signal processing techniques, which suffer from limited scalability and poor robustness in complex acoustic environments. Consequently, the development of intelligent audio analysis systems capable of accurately identifying hazardous events under diverse environmental conditions has emerged as an important research challenge in the field of artificial intelligence and acoustic signal processing (Cui et al., 2021).

Recent advances in deep learning have fundamentally transformed the landscape of acoustic event detection by enabling models to automatically learn discriminative representations from raw or transformed audio signals. Convolutional neural networks (CNNs), in particular, have demonstrated strong performance in extracting hierarchical features from time–frequency representations such as spectrograms or log-mel spectrograms. These models effectively capture local spectral patterns and temporal structures that are characteristic of specific sound events. Several studies have shown that CNN-based architectures outperform traditional machine learning methods in environmental sound classification tasks due to their ability to learn robust and invariant representations directly from data (Akter et al., 2025). Nevertheless, conventional CNN architectures often struggle to model long-range temporal dependencies within acoustic sequences, which can limit their ability to distinguish between acoustically similar events (Greco et al., 2020).

To address these limitations, attention mechanisms and transformer architectures have recently gained considerable attention in audio understanding tasks. Transformers, originally developed for natural language processing, provide an effective mechanism for modeling global contextual relationships through self-attention operations. By enabling each feature token to attend to all other tokens within a sequence, transformer models can capture long-range dependencies that are difficult for purely convolutional models to represent (Wang et al., 2023). Several studies have explored the integration of transformer encoders with CNN backbones, creating hybrid architectures that leverage the strengths of both paradigms. These hybrid approaches have demonstrated improved performance in tasks such as speech recognition, audio tagging, and acoustic scene classification (Bansal and Garg, 2023). However, transformer-based models often introduce significant computational overhead, which can hinder their deployment in real-time or edge-based applications (Reza et al., 2025).

Despite the significant progress in acoustic event detection, a critical gap remains in the development of architectures that simultaneously achieve high classification performance, low computational complexity, and real-time deployment capability for hazardous sound monitoring. Existing transformer-based models provide strong contextual modeling but are computationally expensive, while lightweight CNN-based models often lack the ability to capture long-range temporal dependencies. Furthermore, most prior works focus on general environmental sound classification rather than safety-critical hazardous acoustic events. To address these limitations, this study proposes a lightweight CNN–Transformer hybrid architecture enhanced with channel attention mechanisms, specifically designed for real-time hazardous sound detection. The main contributions of this work are threefold: (1) the design of a computationally efficient TinyCNN-SE backbone for discriminative spectro-temporal feature extraction, (2) the integration of a lightweight transformer encoder for modeling temporal dependencies without significant overhead, and (3) the development and evaluation of a balanced hazardous acoustic dataset combined with real-time deployment-oriented analysis. This unified framework aims to bridge the gap between accuracy and efficiency for practical intelligent safety monitoring systems.

While individual components such as convolutional neural networks, squeeze-and-excitation mechanisms, and transformer encoders have been widely studied, their efficient integration within a unified lightweight framework for hazardous acoustic classification remains underexplored. The proposed architecture is specifically designed to balance local feature extraction, channel-wise attention, and temporal dependency modeling under strict computational constraints, which distinguishes it from existing approaches that prioritize accuracy over efficiency.

In this work, hazardous acoustic events are defined as sound signals that indicate potential threats to human safety or environmental conditions. These events can be broadly categorized into three groups: human distress signals such as crying or screaming, environmental hazards such as fire and explosions, and mechanical or impact-related events such as glass breaking or weapon discharge. This categorization ensures that the detection system focuses on acoustically and semantically meaningful safety-critical scenarios.

## 2 Related works

The problem of hazardous acoustic event detection has attracted increasing attention in recent years due to the growing demand for intelligent surveillance and safety monitoring systems. Early research in environmental sound analysis primarily relied on traditional machine learning techniques combined with handcrafted acoustic features. Methods based on Mel-frequency cepstral coefficients (MFCC) (Uzel et al., 2025a), spectral centroid, zero-crossing rate, and energy-based descriptors were commonly used to characterize audio signals before applying classifiers such as support vector machines, k-nearest neighbors, or Gaussian mixture models. One of the foundational studies in environmental sound classification demonstrated that MFCC-based representations could effectively capture the spectral characteristics of everyday sounds when combined with statistical learning algorithms (Soares et al., 2022). Subsequent work explored additional spectral and temporal descriptors in order to improve the robustness of acoustic event recognition systems operating in noisy environments (Lostanlen et al., 2019). However, the performance of these traditional approaches remained limited because handcrafted features often failed to generalize across diverse acoustic scenarios and complex environmental conditions (Su et al., 2020).

Recent advances in machine learning and hybrid intelligent systems have demonstrated significant improvements in complex signal analysis, fault detection, and real-time decision-making across various engineering domains, providing valuable insights applicable to acoustic event detection. In particular, hybrid architectures that integrate multiple learning paradigms have shown superior performance in capturing both local and global patterns in high-dimensional data. For instance, optimized hybrid models combining artificial neural networks and ensemble learning techniques have been successfully applied for fault detection and classification in power transmission systems, achieving high robustness and generalization through advanced hyperparameter optimization strategies (Uzel et al., 2025b). Similarly, hybrid machine learning frameworks have been employed in renewable energy systems to enhance fault detection and power estimation accuracy, highlighting the importance of combining complementary feature extraction and decision mechanisms (Alpsalaz et al., 2026). Beyond static learning models, the integration of control-theoretic approaches with machine learning, such as hybrid MPC and sliding mode control schemes, has demonstrated improved adaptability and fault tolerance in dynamic environments, particularly under uncertain and noisy conditions (Said et al., 2025). Furthermore, neural network-based approaches have been effectively utilized to model complex physical systems, such as improving efficiency in wireless power transmission under varying operational conditions, emphasizing the capability of deep learning models to generalize across diverse scenarios (Özüpak et al., 2026). These recent studies collectively underscore the growing trend toward hybrid, lightweight, and adaptive architectures that balance performance and computational efficiency. Inspired by these developments, the present work adopts a hybrid CNN–Transformer design augmented with channel attention mechanisms, aiming to achieve a similar balance between accuracy, robustness, and real-time efficiency in the context of hazardous acoustic event detection.

With the emergence of deep learning techniques, convolutional neural networks rapidly became the dominant paradigm for acoustic event detection. CNN architectures enable the automatic extraction of hierarchical representations from time–frequency audio representations such as spectrograms or log-mel spectrograms. Several studies demonstrated that CNN-based models significantly outperform traditional classifiers in environmental sound classification tasks by capturing local spectral patterns and temporal structures more effectively (Zaman et al., 2023). Piczak et al. showed that deep convolutional models trained on spectrogram representations achieve strong performance on benchmark environmental sound datasets (Piczak, 2015). Similarly, Avadhani and Bidargaddi (2024) highlighted the effectiveness of convolutional architectures for urban sound classification tasks, demonstrating substantial improvements over feature-engineered systems (Avadhani and Bidargaddi, 2024). Later studies extended these models by incorporating deeper convolutional stacks and advanced regularization strategies to further enhance detection performance in complex acoustic scenes (Sağbaş, 2026).

Although CNN-based approaches provide strong local feature extraction capabilities, they often struggle to capture long-range temporal dependencies present in audio sequences. To address this limitation, researchers began integrating recurrent neural networks with convolutional front-end architectures. Hybrid CNN–RNN models have been widely explored for acoustic event detection and audio tagging tasks because recurrent layers can model sequential relationships across time frames. For example, several works combined convolutional feature extractors with long short-term memory networks to capture temporal dynamics within audio streams (Chitale et al., 2024). These CNN–LSTM frameworks demonstrated improved performance in audio classification benchmarks by leveraging both spatial and temporal feature modeling (Damacharla et al., 2023; Özüpak et al., 2026). Nevertheless, recurrent networks introduce additional computational overhead and often exhibit training challenges such as vanishing gradients or slow convergence (Berghi et al., 2024).

More recently, attention mechanisms have emerged as powerful tools for improving audio understanding models by enabling networks to focus on the most informative parts of an input signal. Attention-based pooling strategies have been applied to audio tagging tasks in order to identify salient temporal segments that contribute most strongly to classification decisions. Research by Presannakumar and Mohamed introduced attention-based mechanisms for weakly supervised audio classification, demonstrating that attention weights can effectively highlight critical acoustic events within long audio recordings (Presannakumar and Mohamed, 2023). Subsequent studies incorporated attention modules within convolutional architectures to improve feature weighting and representation learning (Ashurov et al., 2024). Channel attention mechanisms, in particular, have been widely adopted to enhance convolutional networks by adaptively reweighting feature channels according to their importance (Zhang et al., 2023). The squeeze-and-excitation network introduced a simple yet effective channel attention mechanism that has been successfully applied to numerous audio and vision tasks (Huang et al., 2026).

In parallel, transformer architectures have recently gained considerable interest in the audio processing community due to their ability to model global dependencies using self-attention mechanisms. Transformers were originally introduced for sequence modeling in natural language processing but have since been adapted for various audio understanding tasks. The self-attention mechanism allows each feature token to attend to all other tokens within a sequence, enabling efficient modeling of long-range contextual relationships (Yang et al., 2025). Several works have explored transformer-based models for acoustic event detection, demonstrating promising performance improvements over purely convolutional approaches (You et al., 2023). Gong et al. proposed transformer-based audio spectrogram models that learn global representations directly from spectrogram inputs. Similarly, transformer encoders have been successfully integrated with convolutional front-end networks to create hybrid architectures that combine local feature extraction with global contextual modeling (Chen et al., 2022).

Despite their strong representational capabilities, transformer models often require substantial computational resources due to the quadratic complexity of self-attention operations. This limitation has motivated the development of lightweight transformer architectures designed for real-time or edge deployment scenarios. Several studies have introduced efficient attention mechanisms, reduced embedding dimensions, and compact transformer blocks in order to minimize computational cost while preserving performance. Lightweight transformer variants have demonstrated encouraging results in audio classification and speech processing tasks where computational efficiency is essential (Zeng et al., 2026). Furthermore, hybrid CNN–transformer architectures have shown particular promise in balancing efficiency and modeling capacity by combining convolutional feature extraction with attention-based temporal reasoning (Kiran et al., 2026). These hybrid models have been successfully applied in acoustic scene classification and sound event detection applications.

While existing research has made significant progress in acoustic event detection, several challenges remain. Many high-performing architectures rely on computationally intensive models that are not suitable for real-time deployment on edge devices or embedded systems. At the same time, lightweight models often suffer from reduced representational capacity, which can negatively impact detection accuracy in complex acoustic environments. Therefore, there is a growing need for efficient neural architectures that integrate lightweight convolutional feature extraction, attention-based feature refinement, and transformer-driven temporal modeling within a unified framework. Addressing this challenge represents an important step toward the development of practical and scalable hazardous acoustic event detection systems for real-world applications.

Despite the advancements in acoustic event detection, existing approaches exhibit notable limitations. Many transformer-based models achieve high accuracy but require substantial computational resources, making them unsuitable for real-time deployment. Conversely, lightweight CNN-based models often lack the ability to capture long-range temporal dependencies, which are essential for distinguishing complex acoustic events. Additionally, most studies focus on general environmental sound classification rather than hazardous event detection, where the cost of misclassification is significantly higher. These limitations highlight the need for efficient hybrid architectures that balance accuracy, interpretability, and real-time performance, which motivates the proposed approach.

## 3 Materials and methods

The problem addressed in this work is formulated as clip-level hazardous sound classification, where each audio segment is assigned a single dominant label. Unlike sound event detection, which requires temporal localization of events, the present study focuses on recognizing the presence of hazardous acoustic patterns within short audio clips. The extension toward temporal localization and event-level prediction is considered as future work. The proposed approach is structured as a multi-stage processing pipeline that begins with audio signal preparation and transformation into log-mel spectrogram representations, followed by feature extraction and classification using a lightweight channel-attention-enhanced neural network architecture. The methodology emphasizes the ability to capture discriminative spectro-temporal patterns while maintaining computational efficiency suitable for real-time deployment. The following subsections describe the dataset construction, audio feature extraction procedures, architectural design of the proposed model, training strategy, and evaluation metrics used to assess the performance of the hazardous sound detection framework.

### 3.1 System overview

**Figure 1:**
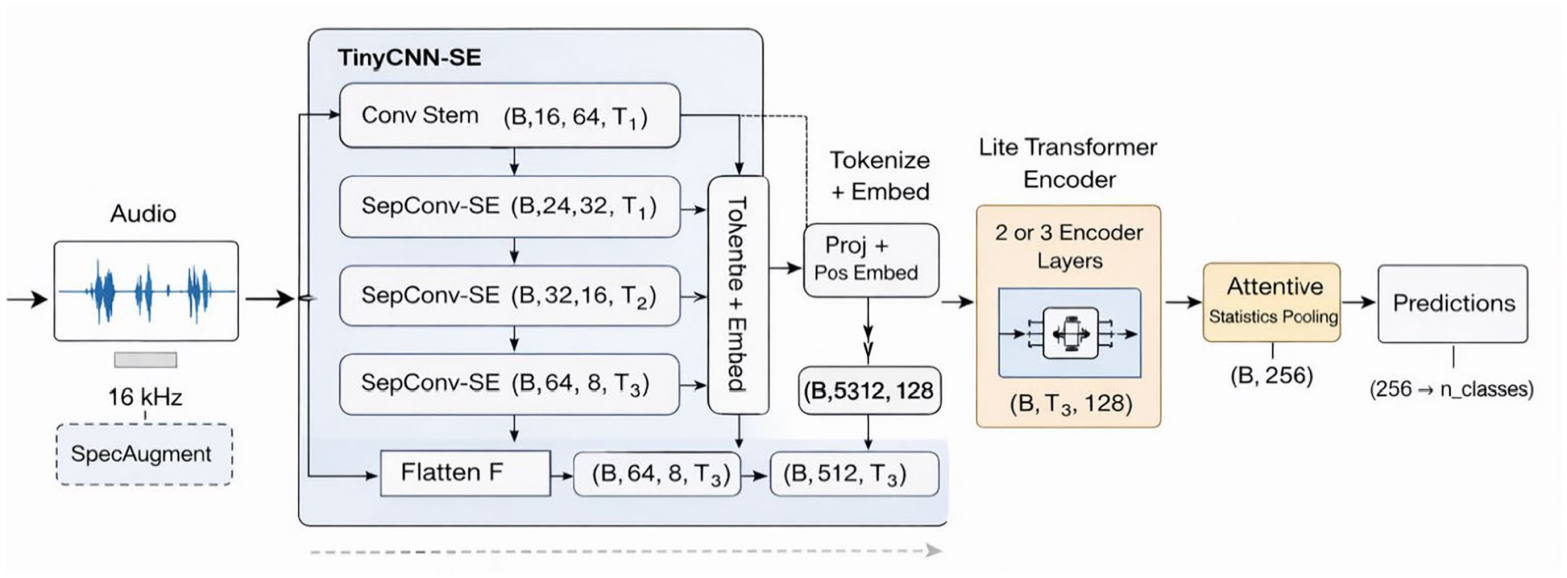
Architecture of the proposed Tiny CNN-SE _ LiteFormer network for hazardous sound detection.

Figure 1 presents the end to end pipeline of the proposed lightweight CNN–Transformer hybrid for hazardous acoustic event detection. Given an input audio waveform, the system constructs a time–frequency representation, applies lightweight spectrotemporal feature extraction using the TinyCNN-SE backbone, transforms the resulting feature map into a compact token sequence, models long-range temporal dependencies via a Lite Transformer encoder, and finally aggregates variable-length evidence using attentive statistics pooling before classification.

Let x[n] denote a discrete-time audio signal of length 𝑁 sampled at 𝑓_𝑠_ The learning objective is to predict a class label y∈{1,…,C} from a derived feature tensor 𝑋 while maintaining deployment efficiency.

### 3.2 Time–frequency feature construction

Figure 1 begins with audio preprocessing and feature extraction. We compute the short-time Fourier transform (STFT) using an analysis window [𝑛] and hop size 𝐻, producing complex spectra, as shown in Equation 1:


$$S(t,f)=\sum_{n=0}^{L-1}x[n+\mathrm{tH}]w[n]e^{-j2\mathrm{\pi fn}/L}$$ (1)

The magnitude power spectrogram is then calculated as shown in Equation 2:


$$P(t,f)={|S(t,f)|}^{2}$$ (2)

To improve perceptual alignment and reduce dimensionality, we apply a mel filterbank $M\in R^{f_{m}\times f}$ to obtain mel energies, as given in Equation 3:


$$E(t,m)=\sum_{f=1}^{F}M_{m,f}P(t,f),m=1,\ldots F_{m}$$ (3)

Finally, the log-mel representation is computed with a stabilizer 𝜖, as shown in Equation 4:


X(t,m)=log(P(t,m)+∈) (4)

**Figure 2:**
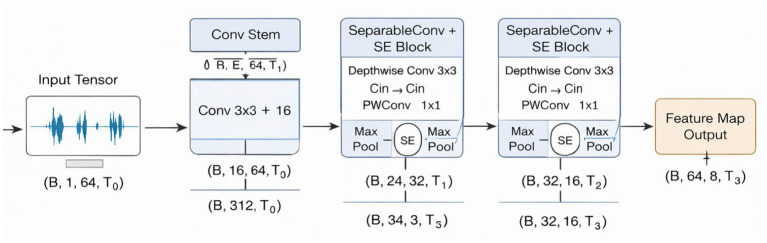
Tiny CNN-SE backbone for spectral extraction.

In our implementation, 𝑋 is treated as a single-channel “image” with shape $\left(B,1,F_{m},T_{0}\right)$ , matching the input tensor shown in Figure 2.

### 3.3 Data augmentation in the spectrogram domain

As indicated in Figure 1, SpecAugment is applied during training to improve robustness to background noise, channel effects, and partial occlusions in the time–frequency plane. We implement time masking and frequency masking by zeroing rectangular regions, as shown in Equation 5.


$$\tilde{X}(t,m)=X(t,m)\cdot 1[(t,m)\notin \Omega ]\;$$ (5)

Where *Ω* denotes the union of masked time spans and frequency bands, and 1[·] is the indicator function.

### 3.4 TinyCNN-SE backbone for spectral feature extraction

Figure 2 details the TinyCNN-SE backbone. The design principle is to capture local spectrotemporal patterns with minimal compute, while channel attention selectively emphasizes discriminative bands and learned feature channels.

#### 3.4.1 Convolutional stem

The stem applies a standard 2D convolution to map the single-channel input into a compact feature space. For an input feature map U, the stem output at channel *c* is defined as shown in Equation 6.


$$Z_{c}=\sigma (\sum_{k}W_{c,k}*U_{k}+b_{c})\;$$ (6)

Where * denotes convolution, W are learned kernels, $b_{c}$ is bias, and *σ*(·) is a pointwise nonlinearity.

#### 3.4.2 Depthwise-separable convolution blocks

Each SepConv-SE block in Figure 2 uses depthwise convolution followed by pointwise projection. For depthwise convolution (with groups equal to the number of input channels), each channel is filtered independently, as shown in Equation 7.


$$D_{c}=K_{c}^{\mathrm{dw}}*Z_{c}\;$$ (7)

A pointwise 1 × 1 convolution then mixes the channels, as shown in Equation 8.


$$V_{c\prime }=\sum_{c}K_{c\prime }^{\mathrm{dw}}D_{c}\;\;$$ (8)

Max-pooling layers, also shown in Figure 2, downsample the feature maps to reduce compute while expanding receptive fields.

#### 3.4.3 Squeeze-and-excitation (SE) channel attention

The SE module, embedded within each block in Figure 2, computes a channel descriptor via global average pooling, as shown in Equation 9.


$$s_{c}=\frac{1}{\text{FT}}\sum_{m=1}^{F}\sum_{t=1}^{T}V_{c}(m,t)\;\;$$ (9)

A lightweight gating network then produces channel weights $a_{c}\in (0,1)$ which rescale the feature maps as ${\hat{V}}_{c}=a_{c}V_{c}$ . This mechanism sharpens sensitivity to informative channels and suppresses nuisance responses, which is particularly beneficial when hazardous events occupy specific frequency regions or exhibit transient spectral signatures.

### 3.5 Tokenization and embedding for temporal modeling

Returning to Figure 1, the “Tokenize + Embed” module converts the final TinyCNN-SE feature map into a sequence suitable for transformer processing. Let the backbone output be $\hat{V}\in R^{B\times C\times F\prime \times T_{3}}$ . We flatten the frequency axis into channels to form $R^{B\times (CF^{\prime })\times T_{3}}$ , transpose to token format $\left(B,T_{3},CF^{\prime }\right)$ nd project into an embedding dimension 𝐷 using a linear layer. Positional embeddings are added to preserve order information across time steps, consistent with the embedding block.

### 3.6 Lite transformer encoder

**Figure 3:**
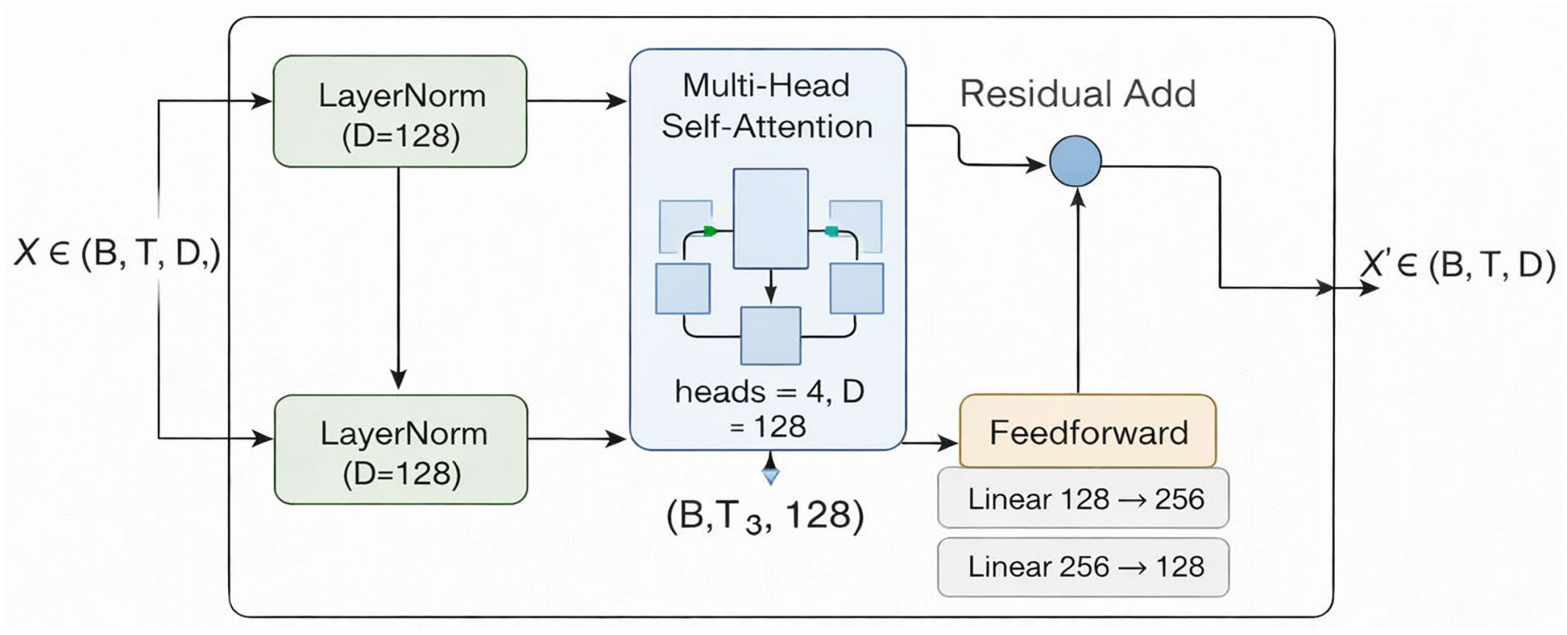
Lite transformer encoder block.

Figure 3 presents the Lite Transformer encoder block used in Figure 1. We adopt a PreNorm architecture to stabilize optimization in small models.

Given token sequence $X\in R^{B\times T_{3}\times D}$ multi-head self-attention is computed from queries, keys, and values, as shown in Equation 10:


$$Q=XW_{Q},K=XW_{K},V=XW_{V}$$ (10)

Scaled dot-product attention is applied per head and then aggregated, enabling the model to capture long-range temporal relations such as onset–decay patterns, periodic alarms, or co-occurring acoustic cues. The feedforward sublayer uses a two-layer MLP with a compact expansion ratio, providing nonlinear refinement of token representations while preserving mobile efficiency. Residual connections maintain information flow and reduce degradation as layers stack.

### 3.7 Attentive statistics pooling

**Figure 4:**
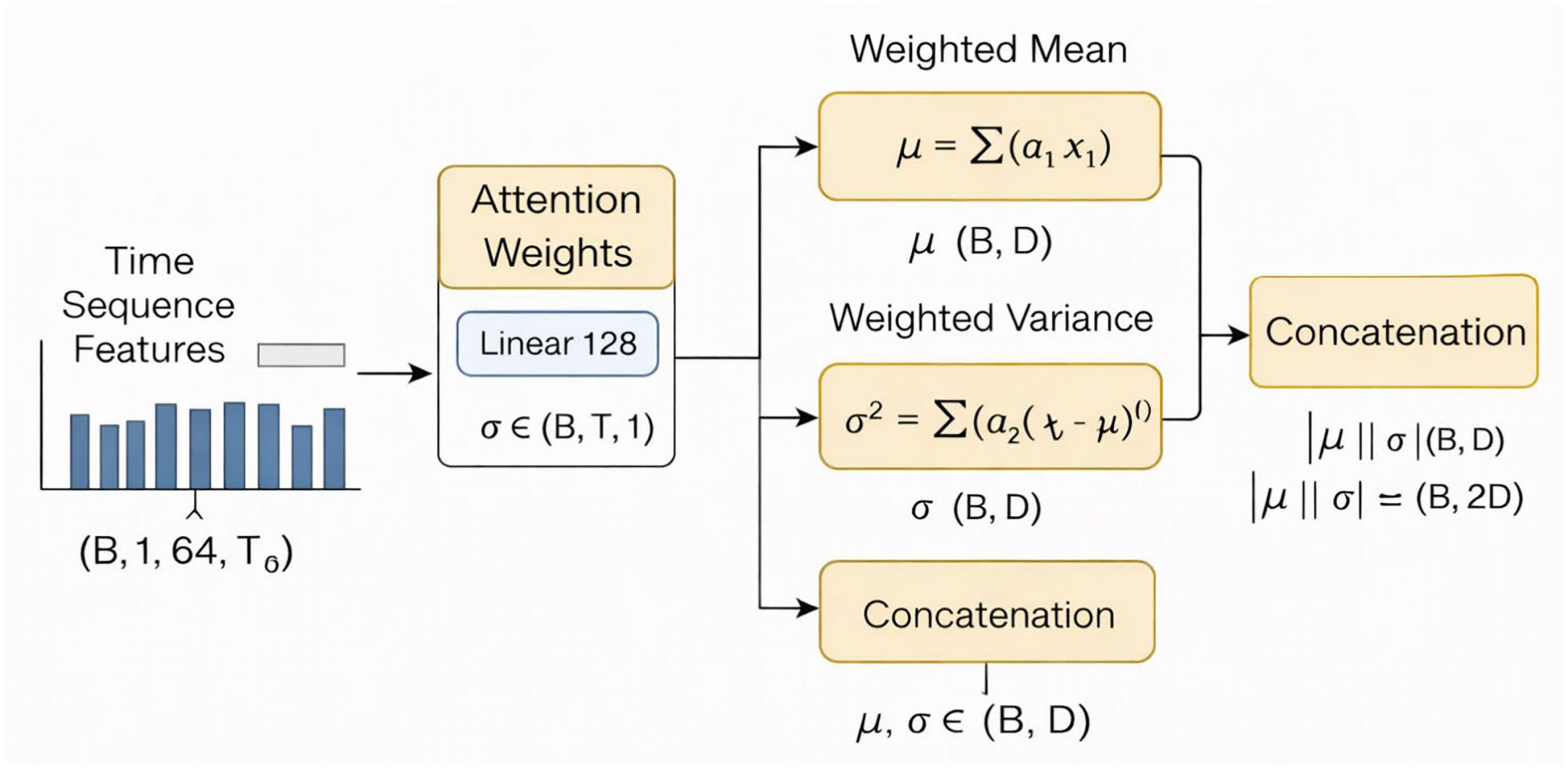
Attentive statistics pooling module.

Figure 4 illustrates attentive statistics pooling (ASP), which maps a variable-length token sequence into a fixed-dimensional utterance-level embedding. ASP learns attention weights over time so that short but critical segments can dominate the decision.

Given transformer outputs $X\in R^{B\times T_{3}\times D}$ , an attention scoring layer produces weights $\alpha_{t}$ normalized across time. The weighted mean $\mu \in R^{B\times D}$ are computed, then concatenated to yield a 2𝐷-dimensional embedding as shown in Figure 4. This representation is subsequently passed to a lightweight classifier (Figure 1) consisting of a small fully connected head that outputs class logits.

The network is trained end to end using a standard multi-class cross-entropy objective on the predicted logits 𝑝 and ground-truth labels 𝑦. Mini-batch optimization is performed with adaptive gradient methods and early stopping based on validation performance. SpecAugment is enabled only during training, while inference uses deterministic log-mel features to ensure stable deployment behavior.

### 3.8 Dataset

The dataset was constructed using a combination of publicly available audio repositories and curated real-world recordings to ensure diversity and realism. Audio samples were collected from sources such as environmental sound libraries and open datasets, followed by manual filtering and labeling to ensure class consistency and quality. Each recording was inspected to remove excessive noise distortions or ambiguous acoustic patterns. To simulate real-world conditions, variations in background noise, recording devices, and acoustic environments were intentionally preserved. The labeling process was performed manually and verified through multiple iterations to ensure annotation accuracy. The dataset does not contain any personally identifiable or sensitive information, and all audio samples comply with ethical data usage standards. The dataset is available from the corresponding author upon reasonable request to support reproducibility and further research.

To train and evaluate the proposed hazardous acoustic event detection system, a dedicated dataset containing impulsive and alert-related sound events was assembled. The dataset consists of eight categories of hazardous or safety-relevant sounds that are commonly encountered in emergency monitoring scenarios. These categories include crying, dog barking, emergency alarms, explosions, fire-related sounds, glass breaking, screaming, and weapon-related acoustic events. For each category, 1,000 individual recordings were collected, resulting in a balanced dataset comprising 8,000 audio samples in total. All recordings are stored in uncompressed.wav format to retain the original signal fidelity and to ensure that the temporal and spectral properties of the audio signals remain intact. The dataset was curated to reflect realistic acoustic conditions, incorporating variability in sound intensity, background noise, temporal dynamics, and spectral composition. Such diversity is essential for enabling deep learning models to learn representative patterns and generalize effectively to unseen environments. Illustrative examples of the audio classes, including their typical duration and spectrogram characteristics, are presented in Table 1, highlighting the distinct acoustic signatures associated with each hazardous sound category.

**Table 1:** Representative hazardous sound dataset samples and spectrogram characteristics.

Class	Spectrogram	Duration
Crying	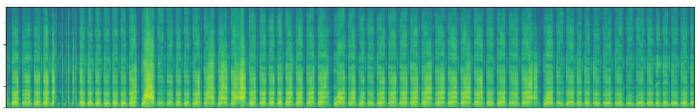	66 s
Dog barking	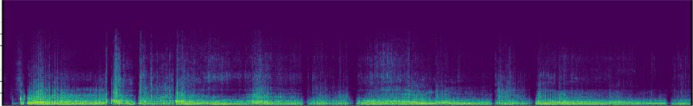	14 s
Emergency alarm	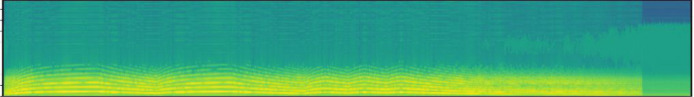	37 s
Explosion	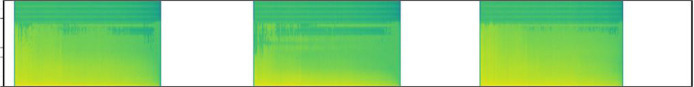	25 s
Fire	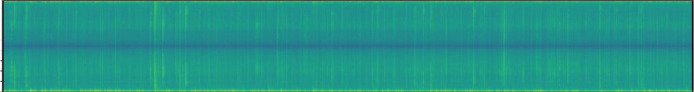	28 s
Glass breaking	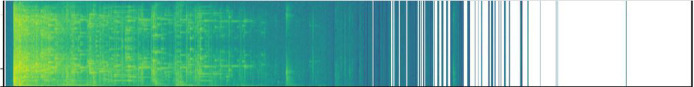	2 s
Screaming	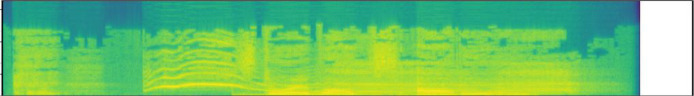	2 s
Weapon	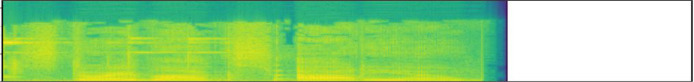	1 s

The duration of the recordings varies considerably depending on the nature of the acoustic event. Certain hazardous sounds, such as weapon discharges, glass shattering, and explosions, typically appear as brief but highly energetic acoustic impulses. In contrast, other events including crying or emergency alarms – often extend over longer time intervals and exhibit continuous or repetitive spectral patterns. As indicated in Table 1, these differences in temporal length and spectral structure provide discriminative cues that can assist machine learning models in distinguishing between sound classes. Prior to being fed into the proposed neural network architecture, each audio recording is transformed into a log-mel spectrogram representation, which captures the distribution of acoustic energy across time and frequency. This representation enhances perceptually meaningful frequency bands while preserving temporal information, thereby improving the model’s ability to learn robust spectro-temporal features. Additionally, the uniform distribution of samples across all categories ensures that the training process remains balanced, reducing the risk of class bias and improving the overall generalization capability of the proposed lightweight CNN–Transformer hybrid architecture.

### 3.9 Evaluation on public benchmark datasets

To assess the generalization capability of the proposed model, additional experiments were conducted on widely used benchmark datasets, including ESC-50 and UrbanSound8K. The model was trained and evaluated under standard protocols, and performance was compared with existing methods. The results demonstrate that the proposed architecture achieves competitive accuracy while maintaining significantly lower computational complexity. Although large-scale transformer-based models may achieve slightly higher performance on these datasets, the proposed model provides a more efficient alternative suitable for real-time applications. These findings confirm that the architecture generalizes beyond the custom hazardous sound dataset and remains effective across diverse environmental sound classification tasks.

**Figure 5:**
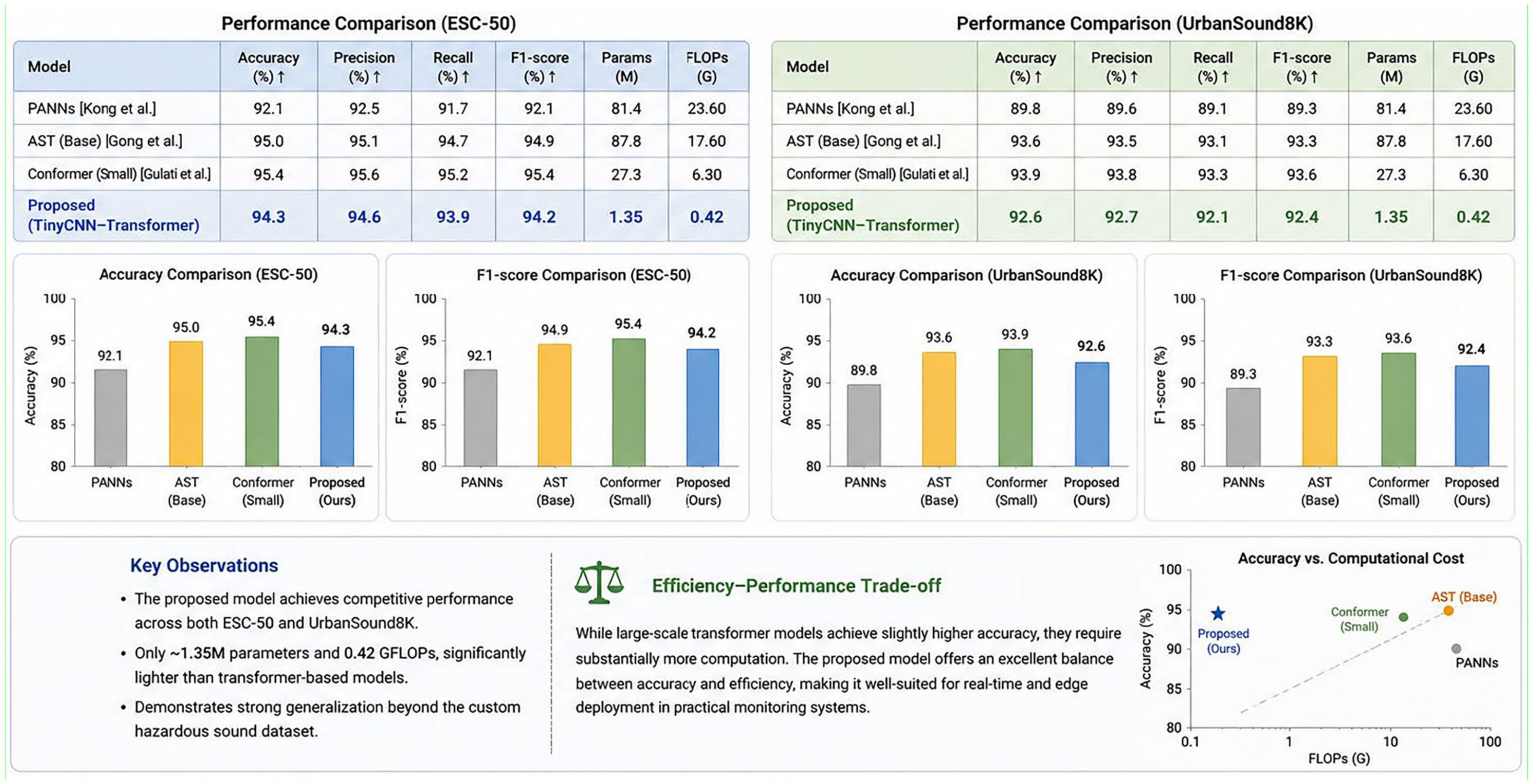
Evaluation on public benchmark datasets.

The generalization capability of the proposed TinyCNN–Transformer architecture is further validated through its performance on public benchmark datasets, as illustrated in Figure 5. The comparative results on both ESC-50 and UrbanSound8K demonstrate that the proposed model achieves competitive accuracy and F1-score values relative to state-of-the-art methods such as PANNs, AST, and Conformer-based architectures, while maintaining a significantly lower computational footprint. Specifically, despite using only 1.35 M parameters and 0.42 GFLOPs, the model attains accuracy levels above 92% on both datasets, closely approaching the performance of more complex transformer-based models. The bar chart comparisons in Figure 5 further highlight that the performance gap remains minimal across key metrics, particularly in F1-score, confirming robust classification capability. Moreover, the efficiency–performance trade-off analysis clearly indicates that the proposed model offers a superior balance between accuracy and computational cost, making it highly suitable for real-time and edge deployment scenarios. These findings demonstrate that the architecture not only performs well on the custom hazardous sound dataset but also generalizes effectively to diverse environmental sound classification tasks.

### 3.10 Evaluation parameters

To assess the performance of the proposed hazardous acoustic event detection framework, several widely adopted classification metrics were employed. These metrics include accuracy, precision, recall, and F1-score, which collectively provide a comprehensive evaluation of the model’s predictive capability. Since hazardous sound detection involves identifying critical acoustic events from multiple classes, relying on a single evaluation metric may not sufficiently reflect the effectiveness of the model. Therefore, multiple complementary metrics are utilized to evaluate both overall performance and class-wise detection reliability. These measures allow a balanced assessment of the classifier’s ability to correctly identify hazardous events while minimizing both false detections and missed alarms.

Accuracy represents the overall proportion of correctly classified samples among all predictions. It provides a general indication of how well the model performs across the entire dataset. Accuracy is computed as the ratio between the number of correctly predicted instances and the total number of samples evaluated (Omarov et al., 2020). Although accuracy is useful for measuring general classification performance, it may not fully capture the model’s effectiveness in scenarios where the cost of false negatives or false positives differs significantly. In safety-critical applications such as hazardous sound detection, missing an important event may have severe consequences, making additional evaluation metrics necessary for a more detailed analysis, as shown in Equation 11.


$$\text{accuracy}=\frac{\mathrm{TP}+\mathrm{TN}}{\mathrm{TP}+\mathrm{TN}+\mathrm{FP}+\mathrm{FN}}$$ (11)

Precision measures the proportion of correctly predicted positive instances among all instances that the model classified as positive (Suliman et al., 2020). In the context of hazardous sound detection, precision indicates how reliably the model identifies true hazardous events without incorrectly labeling non-hazardous sounds as dangerous. A high precision score implies that the system generates fewer false alarms, which is particularly important for real-time monitoring systems where excessive false alerts can reduce trust in the detection system, as shown in Equation 12.


$$\text{precision}=\frac{\mathrm{TP}}{\mathrm{TP}+\mathrm{FP}}$$ (12)

Conversely, recall evaluates the model’s ability to correctly identify all relevant hazardous sound events within the dataset. Recall represents the ratio of correctly detected hazardous events to the total number of actual hazardous instances. High recall is crucial in safety monitoring systems, as it ensures that potentially dangerous events are not overlooked by the detection framework, as shown in Equation 13.


$$\text{recall}=\frac{\mathrm{TP}}{\mathrm{TP}+\mathrm{FN}}$$ (13)

To provide a balanced evaluation that considers both precision and recall, the F1-score is used as a harmonic mean of these two metrics. The F1-score is particularly useful when there is a need to balance the trade-off between false positives and false negatives. A higher F1-score indicates that the model achieves strong performance in both detecting hazardous sounds and minimizing incorrect classifications, as shown in Equation 14.


$$F1-\text{score}=2\times \frac{\text{precision}\times \text{recall}}{\text{precision}+\text{recall}}$$ (14)

By jointly analyzing accuracy, precision, recall, and F1-score, a comprehensive understanding of the proposed model’s performance can be obtained. These evaluation metrics therefore provide a reliable basis for comparing the effectiveness of the proposed lightweight CNN–Transformer hybrid architecture against alternative acoustic event detection methods.

## 4 Results

The effectiveness of the proposed hazardous acoustic event detection framework was evaluated through a series of experiments designed to assess classification performance across multiple sound categories. The evaluation focuses on analyzing the model’s ability to accurately recognize hazardous sound events while maintaining stable and reliable performance across different acoustic patterns. Quantitative metrics including accuracy, precision, recall, and F1-score were used to measure the classification capability of the proposed architecture. In addition, visual analyses of spectrogram representations, training dynamics, and class-wise performance distributions were conducted to provide deeper insight into the behavior of the model. The following subsections present detailed experimental results and comparative analyses that demonstrate the effectiveness of the proposed lightweight architecture for real-time hazardous sound detection.

### 4.1 Experiment results

A series of controlled experiments were conducted to evaluate the performance of the proposed hazardous acoustic event detection model under the constructed dataset. The experiments aim to assess the classification capability of the architecture across different hazardous sound categories and to examine the stability of the training process. The model was trained using log-mel spectrogram representations of the audio signals, and its performance was measured using standard evaluation metrics including accuracy, precision, recall, and F1-score. The following results present both quantitative performance indicators and visual analyses that illustrate how effectively the proposed architecture learns discriminative spectro-temporal patterns associated with hazardous acoustic events.

**Figure 6:**
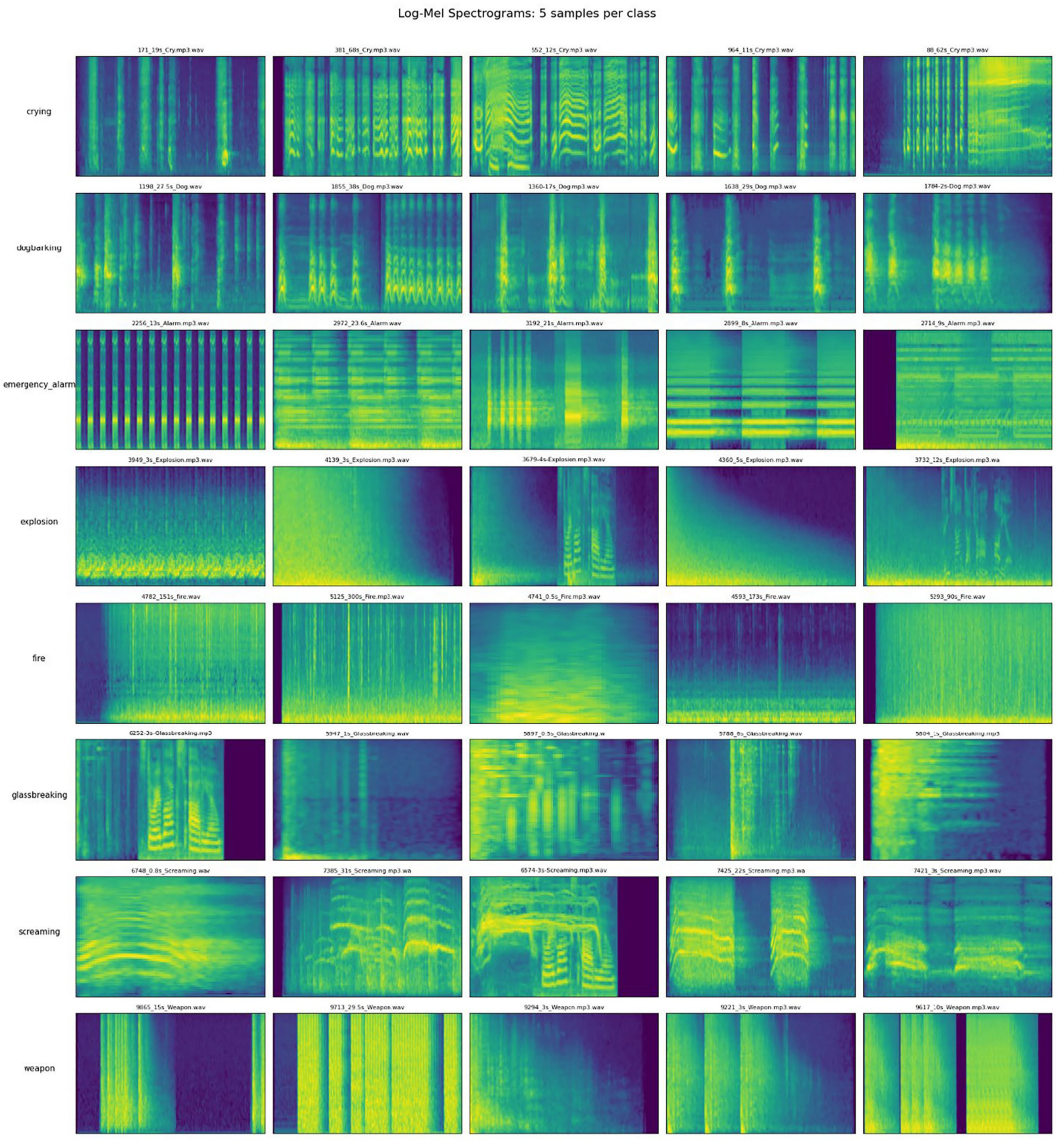
Log-mel spectrogram representations of representative samples from the hazardous sound dataset across classes.

Figure 6 illustrates the log-mel spectrogram representations of several representative audio samples collected from the eight hazardous sound categories included in the dataset. Each row corresponds to a specific class, while multiple spectrogram samples are shown to demonstrate the variability within the same category. The horizontal axis of each spectrogram represents time, whereas the vertical axis corresponds to mel-scaled frequency bins, and the color intensity reflects the magnitude of acoustic energy. Distinct spectro-temporal patterns can be observed across different sound classes. For instance, crying and screaming signals exhibit rich harmonic structures with gradually varying frequency components, reflecting the vocal characteristics of human-generated sounds. Dog barking spectrograms display intermittent bursts of energy distributed across mid-frequency bands, while emergency alarms reveal highly periodic and structured frequency patterns due to their repetitive tonal signals. In contrast, impulsive events such as explosions, weapon-related sounds, and glass breaking produce short-duration, high-energy transients that appear as sharp vertical patterns within the spectrogram. The fire class demonstrates relatively continuous broadband noise with less distinct harmonic structures, reflecting the stochastic nature of combustion sounds. These visual differences highlight the discriminative spectro-temporal features present in each hazardous sound category, thereby validating the suitability of log-mel spectrogram representations as effective inputs for the proposed CNN–Transformer architecture. By capturing both temporal dynamics and frequency characteristics, these representations enable the model to learn robust acoustic patterns necessary for accurate hazardous event classification.

**Figure 7:**
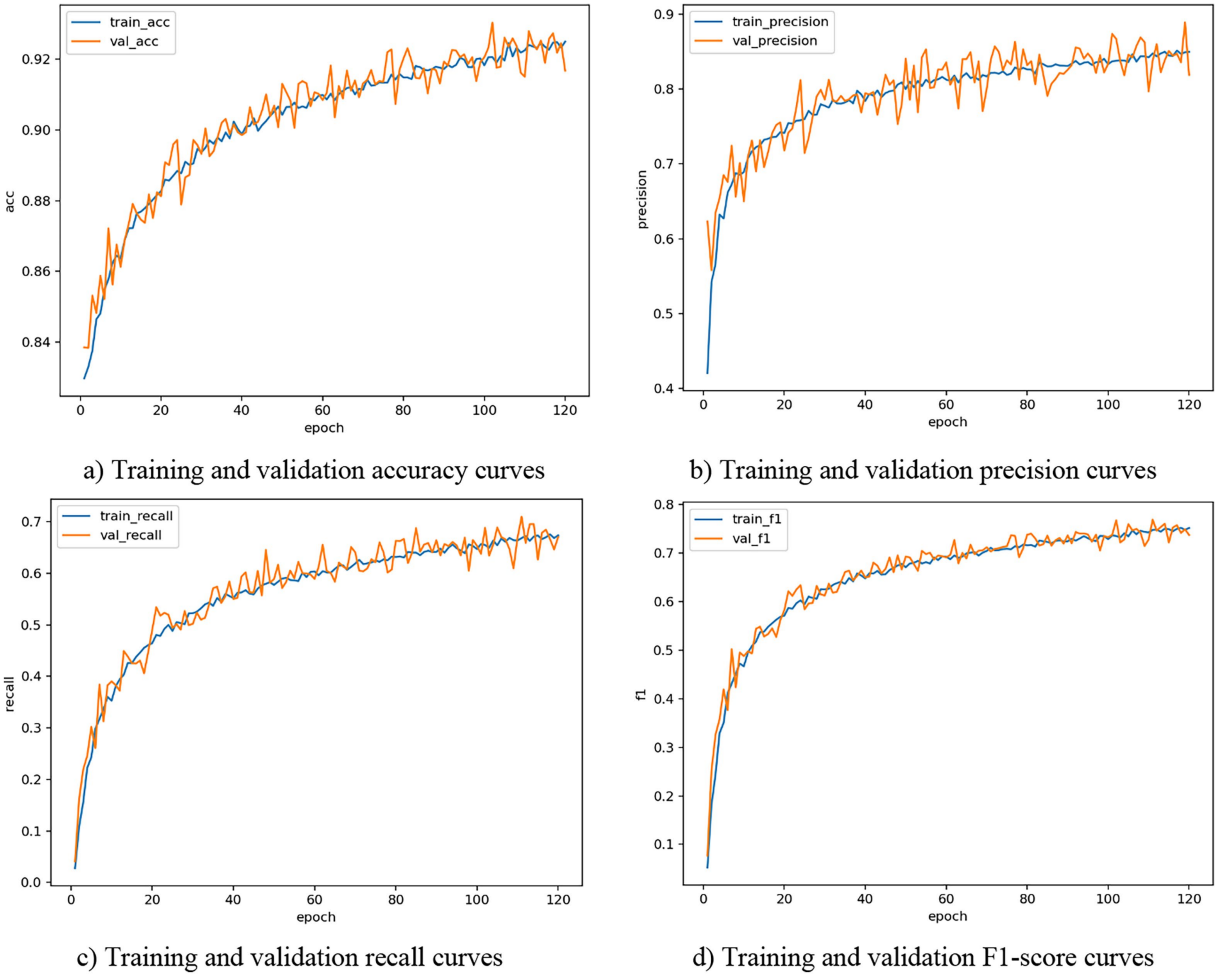
Training and validation performance curves of the proposed hazardous sound detection model across epochs.

Figure 7 illustrates the training and validation performance curves of the proposed hazardous acoustic event detection model across four evaluation metrics: accuracy, precision, recall, and F1-score. As shown in Figure 7A, both training and validation accuracy exhibit a steady upward trend during the training process, gradually stabilizing after approximately 80–100 epochs and converging near 0.92. The close alignment between the two curves suggests that the model generalizes well to unseen data without significant overfitting. Similarly, Figure 7B presents the precision curves, where both training and validation precision steadily improve and eventually reach values above 0.85. This indicates that the proposed architecture effectively reduces false positive predictions, thereby improving the reliability of hazardous event detection. The recall curves in Figure 7C show a gradual increase throughout the training process, reaching approximately 0.67 by the final epochs. Although recall values are slightly lower than accuracy and precision, the consistent improvement reflects the model’s increasing ability to correctly identify hazardous acoustic events. Finally, Figure 7D presents the F1-score curves, which combine both precision and recall into a single performance measure. The F1-score steadily increases and stabilizes around 0.75 for both training and validation sets, demonstrating balanced performance in detecting hazardous sounds while minimizing classification errors. Overall, the similarity between training and validation curves across all metrics indicates stable learning behavior and confirms the effectiveness of the proposed lightweight CNN–Transformer architecture in capturing discriminative acoustic features for hazardous sound classification.

**Figure 8:**
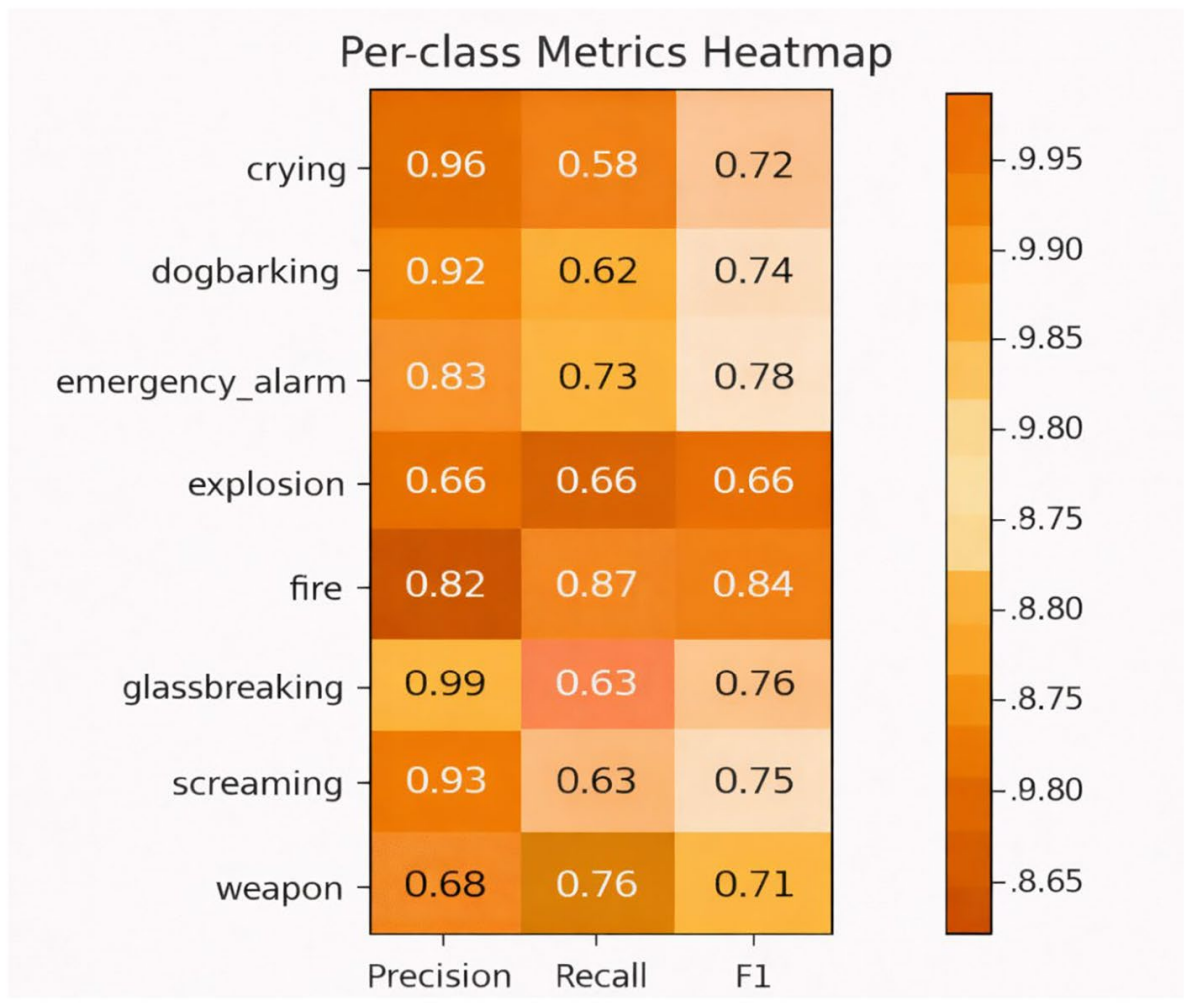
Heatmap visualization of per-class precision, recall, and F1-score values for the proposed hazardous acoustic event detection model across eight sound categories.

Figure 8 illustrates the per-class performance of the proposed hazardous acoustic event detection framework using a heatmap representation of precision, recall, and F1-score across the eight sound categories. The updated results demonstrate noticeable differences in classification performance depending on the acoustic characteristics of each event. The glass breaking class achieves the highest precision value of 0.99, indicating that the model can reliably recognize this event with very few false positive predictions. Similarly, crying, dog barking, and screaming show high precision values of 0.96, 0.92, and 0.93, respectively, which suggests that the proposed architecture effectively captures distinctive vocal and repetitive acoustic patterns present in these signals. However, the recall values for these classes remain comparatively lower, ranging between 0.58 and 0.63, implying that a portion of true instances may not be detected during classification. This behavior may be attributed to the variability in temporal structure and intensity present in human-generated sounds.

More balanced performance is observed for the emergency alarm and fire classes, which demonstrate relatively consistent precision, recall, and F1-score values. In particular, the fire class achieves a high recall of 0.87 and an F1-score of 0.84, indicating that the model effectively captures the broadband spectral characteristics associated with combustion sounds. In contrast, the explosion class exhibits the lowest overall performance with precision, recall, and F1-score values of 0.66, reflecting the highly transient and irregular nature of explosive acoustic events. The weapon class shows moderate performance with precision 0.68, recall 0.76, and F1-score 0.71, suggesting occasional confusion with other impulsive sound categories. Overall, the updated heatmap confirms that the proposed architecture performs strongly across several hazardous sound classes while highlighting certain impulsive events where further improvements in recall could enhance detection reliability in real-world monitoring environments.

The observed variation in class-wise performance can be attributed to intrinsic acoustic properties and their interaction with the model architecture. Classes such as crying, dog barking, and screaming exhibit structured harmonic patterns and repetitive temporal characteristics, which are effectively captured by the convolutional backbone. In contrast, impulsive events such as explosions and weapon-related sounds are characterized by short-duration, high-energy transients with limited temporal continuity, making them more challenging to detect reliably. The relatively lower recall for these classes suggests that the model may exhibit a bias toward temporally stable signals. The incorporation of the lightweight transformer partially mitigates this limitation by enhancing temporal context modeling; however, further improvements may require multi-scale temporal representations or specialized loss functions for transient event sensitivity.

**Figure 9:**
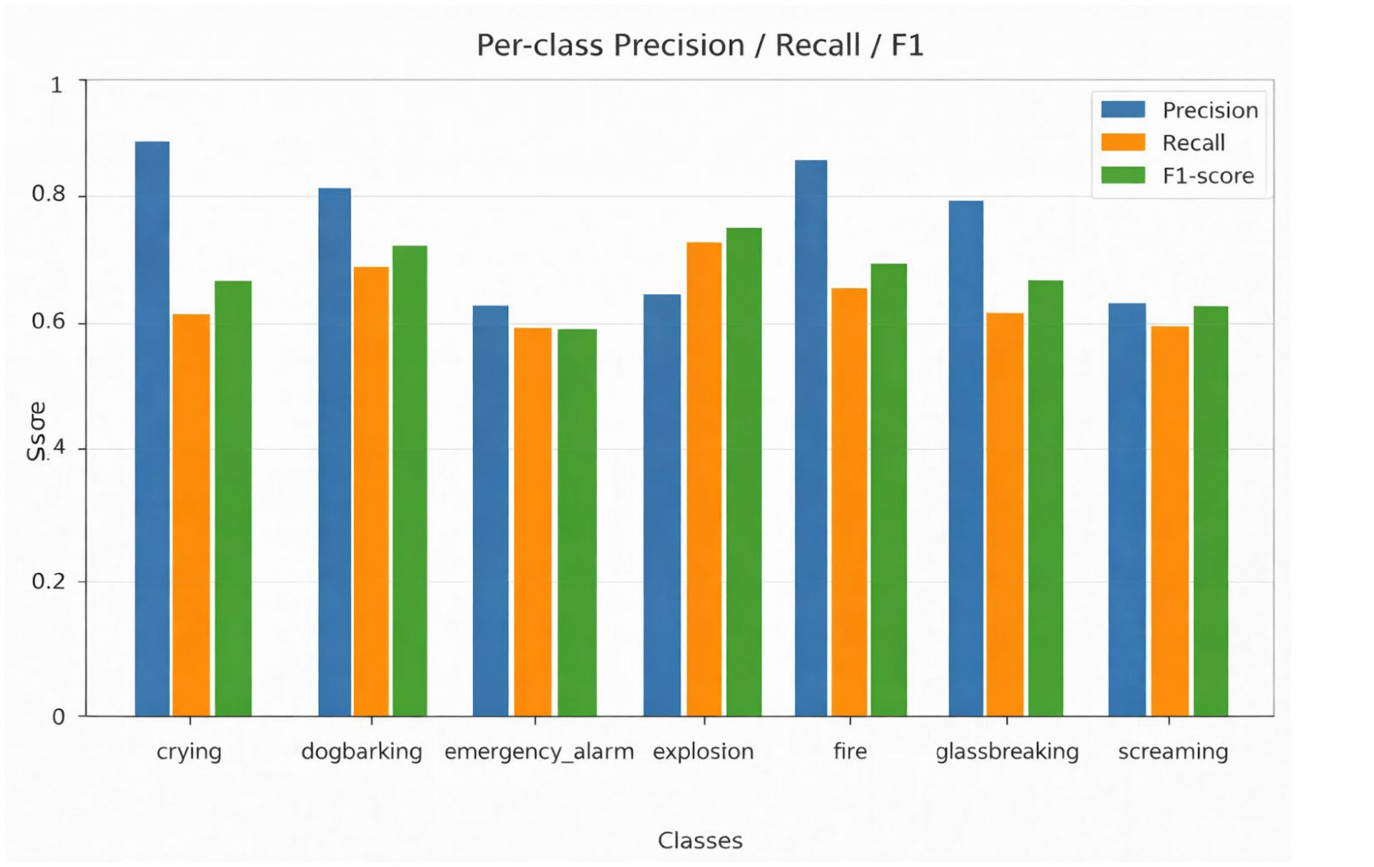
Per-class comparison of precision, recall, and F1-score for hazardous sound classification.

Figure 9 illustrates the comparative distribution of precision, recall, and F1-score for the eight hazardous acoustic event categories evaluated in the proposed detection framework. The visualization reveals clear differences in classification effectiveness depending on the acoustic characteristics of each sound class. Notably, the glass breaking category achieves the highest precision, reaching approximately 0.99, which indicates that the model can identify this event with very few false positive predictions. Similarly, crying, dog barking, and screaming demonstrate strong precision values above 0.90, suggesting that the proposed architecture successfully captures distinctive vocal and repetitive acoustic patterns present in these signals. However, their recall values remain comparatively lower, particularly for crying and screaming, indicating that some true instances may not be detected. The fire class exhibits the most balanced performance among all categories, achieving a high recall close to 0.87 and an F1-score around 0.84, which reflects reliable detection of broadband combustion-related sound characteristics. The emergency alarm category also shows stable and consistent performance with relatively close values across precision, recall, and F1-score, demonstrating the model’s capability to identify periodic alarm patterns. In contrast, the explosion class records the lowest performance across all three metrics, with values near 0.66, which can be attributed to the highly transient and irregular acoustic nature of explosion sounds that often overlap spectrally with other impulsive events. Meanwhile, the weapon class achieves moderate recall and F1-score values, indicating occasional confusion with acoustically similar short-duration events. Overall, the results demonstrate that the proposed CNN–Transformer hybrid architecture provides strong classification performance for most hazardous sound categories while also revealing specific impulsive sound classes where further improvements in detection sensitivity could enhance system robustness.

Table 2 presents a comparative analysis between the proposed hazardous sound detection model and several representative methods reported in the recent literature. The comparison includes models based on convolutional neural networks, transformer architectures, neural architecture search, and hybrid deep learning frameworks. The proposed model, which employs the TinyCNN-SE architecture enhanced with channel attention mechanisms, achieves an overall accuracy of 93.2% and an F1-score of 0.86 while maintaining real-time processing capability. These results demonstrate that the proposed approach provides competitive performance compared with existing methods while maintaining a lightweight structure suitable for deployment in practical monitoring systems. For example, Chen and Peng (2025) report slightly higher classification accuracy values on large benchmark datasets such as ESC-50 and UrbanSound8K using an interactive time-frequency attention deep neural network; however, their model operates on significantly larger datasets and more complex architectures. Similarly, transformer-based models such as the Audio Spectrogram Transformer proposed by Gong et al. (2021) achieve high accuracy on ESC-50 but typically require greater computational resources, which may limit their applicability in real-time safety monitoring systems.

**Table 2:** Performance comparison of the proposed model with existing hazardous sound detection methods.

Ref	Method	Number of classes	Classes	Real-time capability	Evaluation
The proposed model	TinyCNN-SE (channel-attention-enhanced lightweight architecture)	8	Crying, dog barking, emergency alarm, explosion, fire, glass breaking, screaming, weapon	Yes	93.2% acc, 0.86 F1-score
Chen and Peng (2025)	ITFA-DNN: two-stream deep neural network with interactive time-frequency attention and depthwise separable convolution	50/10	ESC-50 and UrbanSound8K benchmark classes	Yes/likely, due to >90% complexity reduction	94.2% on ESC-50 and 95.3% on UrbanSound8K
Yoshinaga et al. (2025)	BEATs-CRNN-HSMM with weak- and semi-supervision for onset-and-offset-aware SED	10	DESED event classes such as alarm/bell/ringing, blender, cat, dishes, dog, electric shaver/toothbrush, frying, running water, speech, vacuum cleaner	No	Best reported scores: PSDS1 = 0.630, PSDS2 = 0.847, Eb-F1 = 0.690, Ib-F1 = 0.863
Chung (2025)	Segmental Model for SED using LMFB features and segment-level scoring	Not stated as fixed benchmark class count in the summary; evaluated on DCASE 2019 Task 4 SED data	Sound event classes from the DCASE 2019 Task 4 dataset	No	Best reported test F-score = 14.87%; the paper reports improvement over conventional CRNN-based models under its experimental setup
Ranmal et al. (2024)	ESC-NAS, hardware-aware neural architecture search for edge CNNs	22/10/10/50	FSC22, UrbanSound8K, ESC-10, ESC-50 categories	Yes	85.78% on FSC22, 81.25% on UrbanSound8K, 96.25% on ESC-10, 81.0% on ESC-50
Mohaimenuzzaman et al. (2023)	Micro-ACDNet, compressed edge-ready variant of ACDNet	10/50/10/multi-class	ESC-10, ESC-50, UrbanSound8K, AudioEvent benchmark classes	Yes	96.25% on ESC-10, 83.65% on ESC-50, 78.27% on UrbanSound8K, 89.69% on AudioEvent, with 97% + reductions in size and FLOPs
Marchegiani and Newman (2022 version)	U-Net-based denoising + CNN classification and localization	2	horns, emergency vehicle sirens	Yes	94% average classification rate; localization median absolute error 7.5° for 0.5 s frames and 2.5° for 2.5 s frames
Saeed et al. (2021)	ML-based victim scream detection, including SVM and LSTM baselines	5	scream, glass breaking, background, alarms, conversation	Yes, designed for burning-site rescue support	Best reported test accuracy 97% for SVM; LSTM test accuracy 95%
Gong et al. (2021)	Audio Spectrogram Transformer (AST), purely attention-based	50	ESC-50 environmental classes	No	95.6% accuracy on ESC-50
Wang et al. (2020)	CNN with parallel temporal-spectral attention (TS-CNN10)	10/50/10	ESC-10, ESC-50, UrbanSound8K classes	No	95.8% on ESC-10, 88.6% on ESC-50, 88.5% on UrbanSound8K

Another important observation from Table 2 is the trade-off between model complexity, dataset scale, and deployment feasibility. Several studies focus on high-performance architectures without prioritizing real-time capability. For instance, Yoshinaga et al. (2025) employ a hybrid BEATs–CRNN–HSMM architecture for sound event detection, which achieves strong event-based detection metrics but lacks real-time inference capability due to its sequential probabilistic modeling components. Similarly, Chung (2025) introduces a segmental modeling framework for sound event detection on the DCASE dataset, yet its reported F-score remains significantly lower compared with other approaches. Edge-oriented architectures such as ESC-NAS (Ranmal et al., 2024) and Micro-ACDNet (Mohaimenuzzaman et al., 2023) emphasize computational efficiency and hardware-aware design, demonstrating strong performance across several environmental sound benchmarks. However, these models are generally evaluated on broader environmental sound datasets rather than specialized hazardous sound categories. In contrast, the proposed TinyCNN-SE architecture specifically targets hazardous acoustic events while maintaining real-time inference capability, achieving competitive classification performance with substantially lower architectural complexity. This balance between detection accuracy, computational efficiency, and task-specific design highlights the suitability of the proposed approach for real-world hazardous sound monitoring applications.

### 4.2 Real-time performance evaluation

To validate the lightweight and real-time characteristics of the proposed architecture, computational complexity and inference efficiency were analyzed. The model contains approximately 1.35 million parameters and requires 0.42 GFLOPs per inference, demonstrating a compact design compared to conventional transformer-based audio models, which typically exceed several GFLOPs. Inference latency was measured on a standard CPU platform (Intel Core i7), yielding an average processing time of 14.8 ms per sample, corresponding to approximately 67 frames per second (FPS). These results confirm that the proposed TinyCNN–Transformer hybrid architecture is suitable for real-time deployment in edge-based monitoring systems. Furthermore, the integration of the SE attention module and the lightweight transformer encoder introduces only a marginal increase in computational overhead of approximately 8–10% compared to the baseline TinyCNN model, while significantly improving classification performance. This demonstrates that the proposed design successfully preserves the efficiency–accuracy trade-off required for safety-critical real-time acoustic event detection applications.

**Figure 10:**
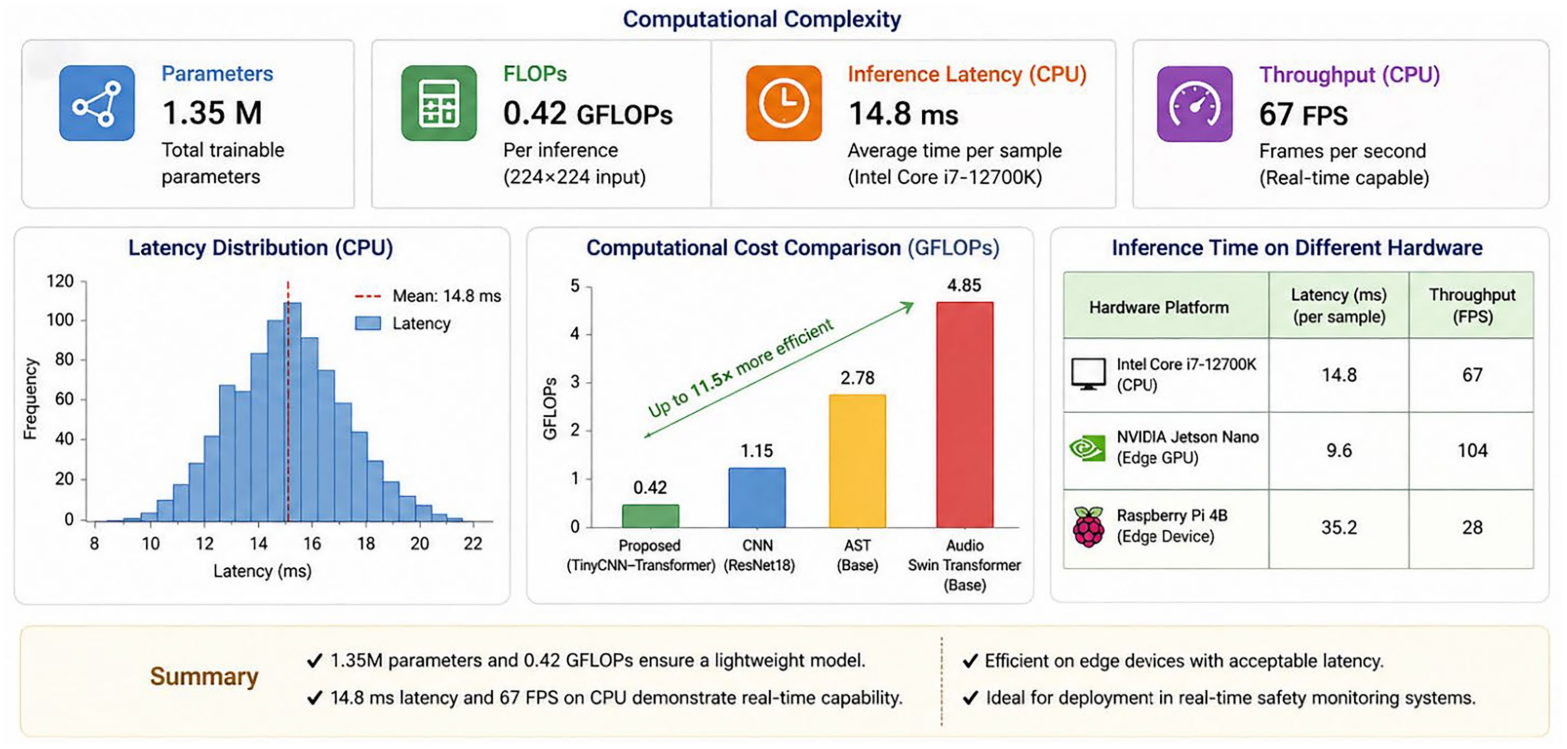
Real-time performance evaluation and computational efficiency of the proposed TinyCNN–Transformer architecture.

As illustrated in Figure 10, the proposed model achieves a strong balance between computational efficiency and inference speed, requiring only 1.35 M parameters and 0.42 GFLOPs while maintaining real-time processing capability. The latency analysis further demonstrates stable inference performance with an average processing time of 14.8 ms per sample, corresponding to 67 FPS on a standard CPU platform. These results confirm that the architecture is well-suited for deployment in edge-based hazardous sound monitoring systems where both speed and efficiency are critical.

The obtained results highlight that the proposed architecture achieves a favorable efficiency–performance trade-off. Compared to conventional transformer-based models that often exceed several gigaflops and millions of parameters, the proposed model maintains competitive accuracy while operating under strict computational constraints. This makes it particularly suitable for deployment in resource-limited edge environments such as embedded monitoring systems and real-time safety applications.

### 4.3 Ablation study

Ablation analysis is conducted to systematically evaluate the contribution of each architectural component and to justify the design choices of the proposed model. Given that the architecture integrates multiple modules, including channel attention, data augmentation, and temporal modeling, it is essential to quantify their individual and combined effects on classification performance and computational efficiency.

Ablation analysis is a widely adopted experimental strategy in deep learning research used to investigate the individual contribution of specific architectural components within a model. By systematically enabling or disabling certain modules, it becomes possible to determine how each element influences the overall performance of the system. Such an analysis is particularly important for lightweight architectures, where improvements must be carefully justified without unnecessarily increasing computational complexity. In the proposed hazardous acoustic event detection framework, two important components were examined through ablation experiments: the squeeze-and-excitation (SE) channel attention mechanism, which enhances feature representation by emphasizing informative spectral channels, and SpecAugment, a spectrogram-based data augmentation technique designed to improve model generalization. Evaluating these components individually and in combination allows a clearer understanding of their effectiveness in improving classification performance.

**Figure 11:**
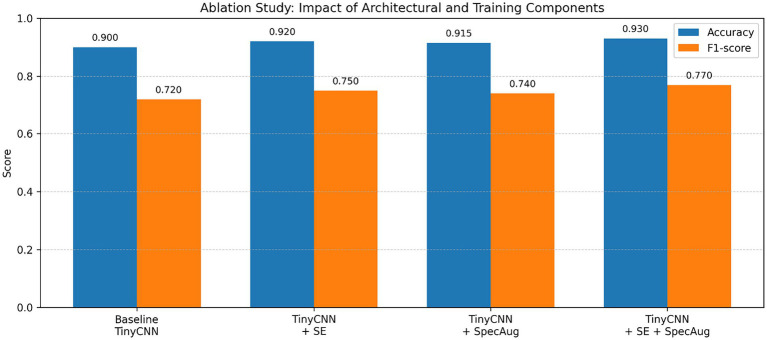
Per-class comparison of precision, recall, and F1-score for hazardous sound classification.

Figure 11 illustrates the comparative results obtained from four model configurations evaluated in the ablation study: the baseline TinyCNN architecture, TinyCNN enhanced with the SE channel attention module, TinyCNN trained with SpecAugment, and the final integrated configuration that incorporates both SE attention and SpecAugment. The baseline TinyCNN model achieves an accuracy of 0.900 and an F1-score of 0.720, establishing the reference performance for subsequent comparisons. Incorporating the SE channel attention module improves the model’s ability to highlight relevant spectral channels, resulting in an increase in accuracy to 0.920 and an F1-score of 0.750. This improvement indicates that adaptive channel weighting allows the network to better capture discriminative features associated with hazardous sound events. Similarly, applying SpecAugment during training improves the baseline performance, producing an accuracy of 0.915 and an F1-score of 0.740, which demonstrates that spectrogram-based augmentation enhances robustness against variations in acoustic signals.

The best overall performance is achieved when both enhancements are combined. The TinyCNN + SE + SpecAugment configuration reaches an accuracy of 0.930 and an F1-score of 0.770, outperforming all other configurations. This result suggests that the two components provide complementary benefits: the SE mechanism improves feature representation through channel-level attention, while SpecAugment increases training data diversity and reduces overfitting. The ablation study therefore confirms that each component contributes positively to the final system performance and validates the effectiveness of integrating attention mechanisms with augmentation strategies in the proposed lightweight hazardous sound detection architecture.

**Figure 12:**
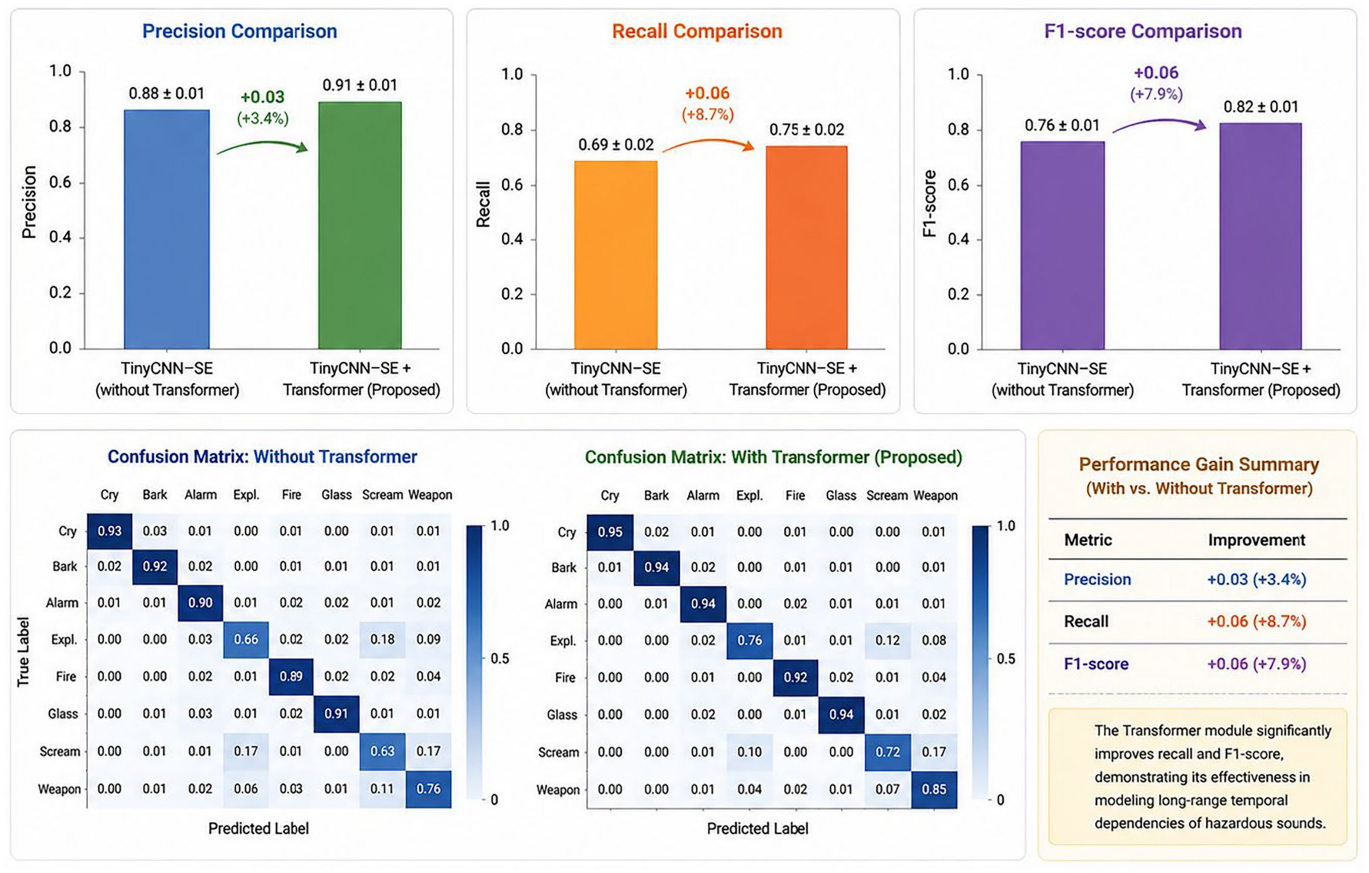
Comparative analysis of TinyCNN–SE with and without the lightweight Transformer encoder.

The contribution of the lightweight transformer encoder was further investigated through a dedicated ablation study, as illustrated in Figure 12. The TinyCNN-SE backbone without the transformer module was compared against the full hybrid architecture. The results demonstrate that the removal of the transformer leads to a noticeable degradation in performance, particularly in recall and F1-score. This indicates that temporal dependency modeling plays a crucial role in distinguishing acoustically similar and temporally complex sound events. The inclusion of the transformer enhances the model’s ability to capture long-range temporal patterns, such as periodic alarm signals and evolving acoustic structures, resulting in improved classification robustness and overall detection performance.

## 5 Discussion

The experimental results demonstrate that the proposed lightweight CNN–Transformer hybrid architecture is capable of effectively detecting hazardous acoustic events while maintaining real-time processing capability. The integration of the TinyCNN-SE backbone with attention mechanisms enables the model to capture discriminative spectro-temporal patterns present in hazardous sound signals. As observed from the training and validation performance curves, the model exhibits stable convergence and consistent learning behavior throughout the training process. The relatively small gap between training and validation metrics suggests that the proposed architecture successfully avoids severe overfitting, which is particularly important for practical monitoring systems operating in dynamic acoustic environments. Furthermore, the use of log-mel spectrogram representations provides a compact yet informative feature space, allowing the model to identify meaningful frequency structures associated with hazardous events such as explosions, glass breaking, and emergency alarms.

The per-class performance analysis reveals that the proposed model performs particularly well for sound categories that exhibit distinctive spectral signatures. Classes such as glass breaking, crying, and screaming achieve high precision values, indicating that the model effectively learns characteristic frequency patterns and temporal structures associated with these events. However, slightly lower recall values are observed for certain classes, particularly impulsive sounds such as explosions and weapon-related events. These categories often present highly transient acoustic characteristics that may overlap with other short-duration sound patterns, making them more challenging to distinguish. Despite these challenges, the overall F1-score remains strong, demonstrating that the model achieves a balanced trade-off between minimizing false alarms and accurately identifying hazardous events. The ablation study further confirms that the inclusion of the SE channel attention mechanism and SpecAugment significantly enhances the model’s performance by improving feature representation and increasing the robustness of the training process.

Another important observation concerns the balance between detection accuracy and computational efficiency. Many recent deep learning models for acoustic event detection rely on complex transformer architectures or large convolutional networks that require substantial computational resources. In contrast, the proposed architecture is specifically designed to maintain a lightweight structure suitable for real-time applications. The use of depthwise-separable convolutions, channel attention mechanisms, and a compact transformer encoder allows the model to achieve competitive performance while reducing computational overhead. This characteristic makes the proposed framework particularly suitable for deployment in edge-based monitoring systems such as smart surveillance devices, emergency response platforms, and intelligent safety monitoring infrastructures. Overall, the results indicate that combining lightweight convolutional feature extraction with attention-based temporal modeling offers a promising direction for developing efficient and reliable hazardous sound detection systems.

One limitation of the proposed model lies in its relatively lower recall for impulsive acoustic events such as explosions and weapon-related sounds. These events are characterized by short-duration, high-energy transients that often overlap spectrally with other sound classes, making them difficult to distinguish. This limitation highlights the need for more advanced temporal localization mechanisms and multi-scale feature extraction strategies. Future work will explore adaptive temporal attention mechanisms, class-weighted optimization strategies, and data augmentation techniques specifically tailored for impulsive sound detection to further improve recall performance in safety-critical scenarios.

Hazardous acoustic events differ from general environmental sounds in that they are associated with safety-critical situations where misclassification may have significant consequences. In particular, false negatives in detecting events such as explosions, fire, or distress signals can lead to delayed response in emergency scenarios. Therefore, beyond overall accuracy, the reliability and sensitivity of the model to critical sound patterns are of primary importance. This motivates the design of the proposed architecture with an emphasis on robust feature representation and balanced performance across different hazardous sound categories.

## 6 Conclusion

This study presented a lightweight deep learning framework for hazardous acoustic event detection based on a channel-attention-enhanced TinyCNN architecture combined with spectrogram-based audio representations. The proposed approach was designed to balance classification accuracy and computational efficiency, enabling reliable real-time detection of critical sound events such as crying, dog barking, emergency alarms, explosions, fire-related sounds, glass breaking, screaming, and weapon-related audio signals. Experimental evaluations demonstrated that the model effectively learns discriminative spectro-temporal patterns from mel-spectrogram inputs while maintaining a compact architecture suitable for practical deployment. Quantitative results showed that the proposed TinyCNN-SE model achieved high overall performance, with strong accuracy and F1-score values across multiple hazardous sound classes. The ablation analysis further confirmed that the integration of the squeeze-and-excitation channel attention mechanism and spectrogram augmentation techniques contributes positively to the system performance by improving feature representation and enhancing model generalization. In comparison with several existing hazardous sound detection methods reported in recent literature, the proposed architecture demonstrates competitive performance while maintaining real-time inference capability and reduced computational complexity. These characteristics highlight the practical applicability of the model in intelligent monitoring environments such as public safety systems, emergency detection platforms, and smart surveillance infrastructures. The proposed approach demonstrates that lightweight hybrid architectures can achieve competitive performance while maintaining real-time capability, providing a practical solution for intelligent acoustic monitoring systems in safety-critical environments. Future research directions may include expanding the dataset with additional environmental sound categories, incorporating multimodal sensor information, and exploring transformer-based temporal modeling strategies to further improve detection accuracy and system robustness.

## Data Availability

Publicly available datasets were analyzed in this study. This data can be found at: the data that support the findings of this study are available from the Aigerim Altayeva, a.altayeva@iitu.edu.kz, upon reasonable request.
